# Clinical Characteristics of Diabetes Mellitus and Suicide Risk

**DOI:** 10.3389/fpsyt.2017.00040

**Published:** 2017-03-13

**Authors:** Chiara Conti, Chiara Mennitto, Giulia Di Francesco, Federica Fraticelli, Ester Vitacolonna, Mario Fulcheri

**Affiliations:** ^1^Department of Psychological, Health, and Territorial Sciences, University “G. d’Annunzio” of Chieti-Pescara, Chieti, Italy; ^2^Department of Medicine and Aging, University “G. d’Annunzio” of Chieti-Pescara, Chieti, Italy

**Keywords:** diabetes mellitus, suicidal behavior, suicidal ideation, suicide attempt, depression

## Abstract

**Background:**

Diabetes mellitus (DM) is a chronic illness with impaired health-related quality of life and a high risk of psychiatric disorders. We carried out a systematic review analyzing the relationship between DM and suicide by providing a qualitative data synthesis of the studies.

**Methods:**

We conducted, in accordance with Preferred Reporting Items for Systematic Reviews and Meta-Analyses guidelines, a systematic search of the literature in PubMed, Scopus, ISI Web of Science, PsycINFO, Google Scholar, and ScienceDirect. Search terms were “suicid*” combined with the Boolean “AND” operator with “diabetes.”

**Results:**

The initial search identified 568 citations. A total of 17 research reports met the predefined inclusion criteria and were analyzed. DM was found to be significantly associated with a marked increase in suicidal behaviors and suicidal ideation (SI), especially in patients with depressive symptoms. Insulin therapy, DM of long duration, and unsatisfactory glycemic control were identified as risk factors for SI in Type 1 (T1DM) and Type 2 (T2DM).

**Conclusion:**

Health-care professionals need to be aware of the higher suicidal risk in patient subgroups based on the clinical characteristics of DM; thus, patients with these characteristics warrant special attention. In this regard, clinical management should include efforts to manage emotional distress in DM care.

## Introduction

Diabetes mellitus (DM) is a metabolic disease characterized by hyperglycemia resulting from defects in insulin secretion, insulin action, or both. The vast majority of cases of diabetes mellitus (DM) falls into two broad etiopathogenetic categories: Type 1 (T1DM) and Type 2 (T2DM) (1). DM is a complex, chronic illness requiring continuous medical care with multifactorial risk-reduction strategies beyond glycemic control (1) to maintain well-being and quality of life over time, to control risks, to manage disease symptoms, and to reduce the incidence of complications (2).

Patients with DM are at risk of physical and psychological complications. The short-term complications include hypoglycemia, and the long-term complications include cardiovascular disease, neuropathy, nephropathy, and retinopathy.

The prevalence of depression and psychiatric diseases among adults with T1DM or T2DM is approximately double that observed in the general population (3, 4).

Depression has a negative impact on self-care (5) and has been shown to be related to poorer glycemic outcomes and therefore increased risk of complications (6).

The presence of psychopathological symptoms, feeling of hopelessness, and fears of chronic nature of the disease may predispose the patient to the risk of suicidal ideations (SIs) and suicidal behavior, but also to neglect health care and lack motivation to adhere to the medical regimen (7). Suicide risk is considered a major psychiatric emergency in patients diagnosed with chronic illnesses (8). DM and particular aspects of living with DM are known to be associated with greater SI (9–12). Chung et al. found that DM was associated with a marked increase in suicidal behaviors (13).

There are few studies on suicidal risk factors in the patient with DM. The risk factors may be related to the patient’s characteristics, such as coping skills, personality profile, additional psychiatric illness including depression and alcohol use, and the feeling of hopelessness. Other risk factors could be a family history of attempted suicide and completed suicide. This is raised by furthermore the chances of suicide are increased by illness-related and situational risk factors such as adverse events, lack of social support, exacerbation of the illness, and gradual increase of DM complications. Another risk factor could be the easy access to means of self-harm by patients with DM (14).

Suicide is one of the highest public health priorities worldwide. The World Health Organization objectives for suicide prevention emphasize identification of high-risk groups (15).

Recognizing the clinical features associated with suicidal risk in patients with DM is crucial in order to realize screening and interventions aimed at prevention that should also take into account SI and suicidal behaviors.

In light of the above, the aim of this manuscript is to provide new insights for performing a systematic review by reporting the most relevant studies analyzing the clinical characteristics of the association between DM and suicide risk.

## Materials and Methods

### Eligibility Criteria

All papers published in English peer-reviewed journals focused on the presence of SI, attempted or committed suicide in patients with T1DM or T2DM with a medical diagnosis were included in the search. Where the title or abstract seems to describe a study eligible for inclusion, the full text was examined to consider its relevance on the basis of the inclusion criteria. Reviews, meta-analyses, commentaries, letters to the editor, books or book chapters, abstracts, and clearly irrelevant papers were excluded. We also excluded abstracts that did not relate to suicide and DM.

### Information Sources and Searches

This review was conducted using the Preferred Reporting Items for Systematic Reviews and Meta-Analyses guidelines (16). The research was carried out in October 2016 on an electronic database: Scopus, ISI Web of Science, PsycINFO, Google Scholar, ScienceDirect, and PubMed were used to identify published studies.

The following keywords were used: “diabetes” AND “suicid*” [Title/Abstract]. After the initial search was performed, the studies were screened for eligibility, duplicates were identified and discarded. The relevant studies were assessed using their titles and abstracts first and, finally, the full review of papers. Studies were discarded when the full text was not available. Results were not limited to chronological age of participants. Searching and eligibility of target responses were carried out independently by two investigators.

## Results

**Figure 1:**
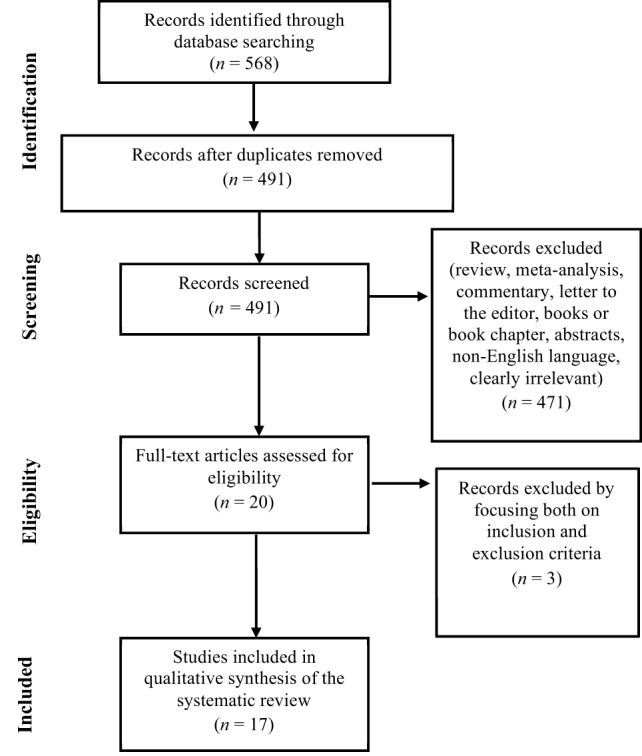
**Preferred Reporting Items for Systematic Reviews and Meta-Analyses Flowchart of the systematic search**.

Based both on inclusion and exclusion criteria, a total of 568 original research studies were identified and selected for inclusion in the systematic review, as reported in the flowchart displayed in Figure 1. After removing the duplicates (*N* = 77) 491 papers remained. After that, reviews, meta-analyses, commentaries, letters to the editor, books or book chapters, abstracts, non-English language, clearly irrelevant papers were eliminated (*N* = 471). Twenty full text articles were assessed for eligibility and were read and surveyed by all the authors. Three articles were excluded because they did not respect the eligibility criteria. At the end of this process of examination, 17 studies were included in the qualitative synthesis of the systematic review.

### Medical Characteristics of Diabetic Patients with Suicide Risk

Studies conducted with diabetic patients have shown significant associations between suicidality and specific diabetes-related characteristics (see Table 1 for a detailed description of reviewed studies). Amer and Hamdan-Mansour (8), by investigating the psychosocial predictors of SI in subjects affected by chronic diseases (T2DM, cardiovascular diseases and cancer), found the highest “seriousness” component among those with T2DM.

**Table 1:** **Distribution of the 17 relevant selected studies, including the reference, title, the population target, and the aims of the research**.

Reference	Title	Population target and geographic location	Aims
Goldston et al. (7)	Suicidal ideation (SI) and behavior and non-compliance with the medical regimen among diabetic adolescents	*N* = 91 Patients with T1DM. United States	To examine the 1 year and lifetime prevalence of SI and suicidal behavior among adolescents with insulin-dependent diabetes mellitus (DM)
Amer and Hamdan-Mansour (8)	Psychosocial predictors of SI in patients diagnosed with chronic illnesses in Jordan	*N* = 442 Patients with T2DM. Jordan	To investigate the psychosocial predictors of SI among patients with chronic illnesses
Pompili et al. (9)	Quality of life and suicide risk in patients with DM	*N* = 100 Patients with T1DM or T2DM. Italy	To evaluate the perceived quality of life and its association with suicide risk in Italian patients with DM
Fuller-Thomson and Sawyer (10)	Lifetime prevalence of SI in a representative sample of Canadians with Type 1 diabetes	*N* = 190 Patients with T1DM. Canada	To compare the lifetime prevalence of SI among patients with and without T1DM
Ceretta et al. (11)	Increased prevalence of mood disorders and SI in Type 2 diabetic patients	*N* = 996 Patients with T2DM. Brazil	To evaluate the association of mood disorders, SI, and the quality of life in patients with T2DM
Handley et al. (12)	Research: educational and psychological issues SI reported by adults with Type 1 or Type 2 diabetes: results from diabetes MILES-Australia	*N* = 3,338 Patients with T1DM or T2DM. Australia	To examine the prevalence and correlates of SI in a community-based sample of adults with DM
Chung et al. (13)	SI and suicide attempts among DM: The Korea National Health and Nutrition Examination Survey from 2007 to 2012	*N* = 3,846 Patients with T1DM or T2DM. Republic of Korea	To evaluate the mental health of patients with DM and compared it with mental health in the general population
Lee et al. (17)	Risk of SI in diabetes varies by diabetes regimen, diabetes duration, and HbA1c level	*N* = 9,159 Patients with T1DM or T2DM. South Korea	To investigate patient subgroups based on the clinical characteristics of DM to evaluate risk factors for SI
Roy et al. (18)	Suicide attempts and ideation in African-American type 1 diabetic patients	*N* = 725 Patients with T1DM. New Jersey	To examine suicidality and its correlates in T1DM and controls through a semi-structured interview on attempted suicide
Davis et al. (19)	Risk of suicide in Australian adults with diabetes: the Fremantle Diabetes Study	*N* = 1,413 Patients with T1DM. Australia	To evaluate the risk of suicide in Australian adult with T1DM
Westling et al. (20)	High CSF-insulin in violent suicide attempters	*N* = 74 DM patients and MDD disorder. Sweden	To further clarify the role of insulin in patients with suicidal behavior
Löfman et al. (21)	Characteristics of suicide among diabetes patients: a population-based study of suicide victims in Northern Finland	*N* = 2,489 Patients with T1DM or T2DM. Finland	To investigate insulin suicides among DM patients
Avci et al. (22)	Suicide commitment with metformin: our experience with five cases	*N* = 5 cases DM patients with metformin treatment. Turkey	To present five patients who used high doses of metformin for suicide attempt
Myers et al. (23)	Brief report: depression and history of suicide attempts in adults with new-onset Type 2 diabetes	*N* = 145 Patients with T2DM. United States	To assess past suicide attempts in a cohort of adults with T2DM diagnosed within the prior 24 months
Han et al. (24)	Increased risk of SI in Korean adults with both diabetes and depression	*N* = 17.065 Patients with T1DM or T2DM. South Korea	To investigate the association between SI and DM in adults with and without depression
Radobuljac et al. (25)	Lifetime prevalence of suicidal and self-injurious behaviors in a representative cohort of Slovenian adolescents with Type 1 diabetes	*N* = 126 Patients with T1DM. Slovenia	To determine lifetime prevalence of suicidal and self-injurious behaviors in adolescents with T1DM compared with healthy controls
Corathers et al. (26)	Improving depression screening for adolescents with Type 1 diabetes	*N* = 528 Patients with T1DM. United States	To evaluate the prevalence of depressive symptoms and SI in a cohort of adolescents with T1DM

Lee et al. (17), in a sample of 9,159 South Korean adults aged 40 years and over, observed a higher rate of SI in subjects with DM than in those without. The study also found an association of SI with the insulin regimen, a longer duration of DM (≥5 years) and a poorer glycemic control (HbA1c levels ≥ 6.5). Another Korean study (13) also reported a marked increase in SI and suicide attempts among participants classified as having DM in comparison with controls. Moreover, mental health problems (depressive mood for two or more continuous weeks, SI, and suicide attempts) increased in association with blood glucose levels.

By focusing on the T1DM population and the risk of suicide attempts, Roy et al. (18) found that African-American T1DM patients were three to four times more likely to attempt suicide during their lifetime than subjects without DM. Davis and colleagues (19), by investigating the suicide risk and associated factors in an older diabetic Australian population, found also that if suicide is a rare event among diabetic adults, compared to the general population, a higher occurrence appeared in subjects with T1DM and a greater diabetes-related disease burden. A bivariate comparison showed also a significant association between suicide with higher presence of retinopathy and greater antidepressant use.

Finally, a previous study (20) measured insulin concentration in cerebrospinal fluid in 74 suicide attempters. The higher cerebrospinal fluid-insulin level detected among patients with a violent suicide attempt than in those with a non-violent attempt, and a similar cerebrospinal fluid–insulin level measured in subjects with or without major depressive disorder, permitted to conclude that cerebrospinal fluid-insulin is involved in violent behavior but not in major depressive disorder.

### Suicide Commitment with Antidiabetic Medications

To confirm the prevalence of T1DM and T2DM in the suicide population and to analyze the suicide method adopted, Löfman et al. (21) conducted a study on a Finnish population. Of all suicide victims (*n* = 2,489) 3.1% had DM (T1DM = 34.6% and T2DM = 65.4%). Almost half of the T1DM victims chosen poison as the suicide method, and it was approximately twofold higher than victims without DM; also in T2DM, self-poisoning was more common compared to controls. Among T1DM victims, an insulin overdose was used by half of the self-poisoning cases (6 of 13), whereas by only two patients in the T2DM group (13%) and none in the non-diabetic reference group. Furthermore, during suicide, half of the victims with T1DM were under the influence of alcohol which, taken excessively, could contribute to the risk of hypoglycemia.

Finally, Avci and colleagues (22) described five cases in which subjects attempted suicide with high doses of metformin, an oral anti-hyperglycemic drug that causes a lactate accumulation. The study also describes the interactions of metformin with other drugs or ethanol that could increase the risk of, or cause, lactic acidosis.

### Suicide Risk and Mood Disorders in DM

Several studies have shown significant associations between DM, mood disorders, and suicidality (see Table 1 for a detailed description of reviewed studies).

In 2009, an Italian study conducted by Pompili et al. (9) observing a sample of 100 patients with T1DM and T2DM controlled for mood disorders found that patients with DM perceived a poor quality of life, which was related to low self-efficacy, high hopelessness, and suicidality.

In 2012, by focusing on a clinical sample consisting of patients with DM and mood disorders, Ceretta et al. (11) demonstrated a high prevalence of depressive disorders, SI, and a poor quality of life in patients with T2DM receiving treatment with insulin.

Löfman and colleagues (21), using a sample of suicide victims, demonstrated the association between DM and suicide mediated by depression.

Myers et al. (23) provided further evidence supporting the prevalence of suicidality and mood disorders in patients with DM by demonstrating that the rate of past suicide attempts in currently depressed patients with DM is 21.8%.

Han et al. (24) investigated, in a large Korean sample, the presence of either DM or depression alone increased the likelihood of SI significantly compared with the general population, by 1.6- and 5.7-fold, respectively. When both conditions were present, the odds of SI were increased more than sevenfold. There was, however, no significant difference in the odds of SI between those with depression only and those with both depression and DM after controlling for factors such as age, gender, and BMI (24).

These results were confirmed by Handley and colleagues in 2015 (12) using a sample of patients with T1DM and T2DM. Particularly, the study showed that elevated rates of SI are largely accounted for by the presence of depressive symptoms, and social support was reported as a significant protective factor for ideations of suicide in these patients.

In order to compare the prevalence of SI in the diabetic South Korean population with that in people without DM, Lee and colleagues (17) compared depressive symptoms, stress, SI, and associated factors using a *chi*-squared test. Depressive symptoms seemed to predict SI, while higher household income, higher education, and being married/cohabiting seemed to play a protective role.

### Suicide Risk in Adolescents with DM

Goldston et al. (7) studied the prevalence of SI and suicidal behavior among adolescents with DM in a sample of 91 outpatients. They found that the lifetime prevalence of SI in diabetic youths was 26.4% (*n* = 24 of 91), which appeared to be higher than the rates of the general population. Some clinical characteristics were associated to SI and suicidal behavior in adolescents with DM. The study, using the multivariate logistic model, showed that the duration of T1DM and the presence of psychiatric problems were related to SI in adolescent patients (7). Furthermore, the authors showed that SI during the previous year was related to non-compliance with medical treatment in 63.6% of the subjects; the authors also found that lifetime history of SI was strongly related to non-compliance with medical treatment in 62.5% of the subjects.

In 2009, a study conducted by Radobuljac et al. (25) found that adolescent females with DM reported a higher prevalence of SI compared to males (*p* < 0.001). The authors studied self-injurious behavior in their sample of 126 patients and observed that 38% of those who committed acts of self-harm used the manipulation of the treatment as a means of self-harm (injecting higher doses of insulin to produce hypoglycemia or omitting insulin to produce hyperglycemia).

Corathers and colleagues (26), using a depression screening tool among patients 13–17 years of age, demonstrated that SI was endorsed in 7% of the sample assessed. In a previous study, Fuller-Thomson and Sawyer (10) had already observed a higher lifetime prevalence of SI among adolescents and adults with T1DM compared to those without, even after adjustment for age and sex; they confirmed the necessity of a consistent screening for SI and depression to promote early identification and intervention in this population.

## Discussion

Literature analysis has shown that the severity of DM can increase the risk of mental health disorders, such as depression and suicide risk. Suicide risk is a multifaceted issue that involves bio-psychosocial and cultural factors that interfere with the individual abilities of patients. Taking the potential clinical implications related to suicide risk in diabetic patients, the present study is a review manuscript aimed at systematically investigating the published original research reports evaluating the emerging clinical links between DM and suicidal factors.

Despite limited and contrasting studies, DM *per se* does not appear to be associated with SI and suicide. However, SI is more prominent among patients with DM than in those without [i.e., Ref. (11, 24, 26)], and this prevalence is associated with the depressive symptoms and the severity of illness, such as duration of DM, poor glycemic control, use of insulin treatment (17). In line with these results, a previous study in 2012 (11) showed that patients with more comorbid conditions were more likely to report depressive disorders and SI versus individuals without DM. Chung et al. (13) further demonstrated that depressive mood, SI, and suicide attempts increased in association with blood glucose levels. Furthermore, in 2004, one study (20) measured insulin concentration in cerebrospinal fluid in 74 suicide attempters to clarify the role of insulin in major depressive disorder and violent suicide attempt and found that cerebrospinal fluid insulin is involved in violent behavior but not in major depressive disorder.

When taking into consideration, the duration of DM as a risk factor for suicide, several studies have investigated the incidence of suicidal behavior (i.e., ideation, attempts, and acts) among adults and elderly patients with T1DM (12, 19). Other studies have highlighted that T1DM patients were three to four times more likely to attempt suicide during their lifetime than subjects without DM (18) and that a higher occurrence appeared in subjects with T1DM and a greater diabetes-related disease burden (10, 19).

Chronic disease causes a major emotional impact, which appears most dangerous during adolescence. When focusing on adolescents with T1DM, a hopeless feeling regarding the illness might be associated with risk of suicide attempts, and also with neglect of health care and lack of motivation to adhere to medical instructions (25). The lifetime prevalence of SI in diabetic youths was 26.4% (7); among patients 13–17 years of age, it was demonstrated that SI was endorsed in 7% of the sample assessed (26).

By focusing on clinical samples consisting of patients with DM and mood disorders, several studies evaluated the association between suicidality and DM. Depressive symptoms were the most prominent predictor of SI in diabetic patients (17). Given the relationships between depression and suicidality, and between depression and DM, some studies examined the mediation role of depressive symptoms, finding that depression mediates the relationship between DM, suicide (21) and non-compliant behavior with the medical regimen (17). In support of these findings, recent studies observed that the coexistence of DM and depression was associated with a much higher risk factor for SI (12, 23) and suicide attempts (13) than with DM alone. In fact, it has also been reported that subjects with an increased prevalence of endocrine abnormalities such as diabetes may be at increased risk of depression and/or anxiety disorders (27). Major depression is also correlated with well-known deficiency in serotonergic neurotransmission, as reported by Müller et al. (28). Neurotransmitter alterations and immune dysregulation lead to an increased generation of proinflammatory cytokines which play a crucial role in anxiety and depression (28, 29). It is interesting that patients with schizophrenia and other severe mental disorders have an increased risk of developing diabetes and hyperlipidemia and initiating medication for these diseases (30).

These findings are important, suggesting that the higher prevalence of suicidal risk observed among people with DM may be attributable to the increased prevalence of depressive symptoms. On the other hand, the study conducted by Pompili et al. (9), observing a sample of patients with DM controlled for mood disorders, found that patients with DM perceived a poor quality of life, which was related to low self-efficacy, high hopelessness, and suicidality. In other terms, the results indicate that DM is associated with an increased risk of suicide, independently from the severity of depressive condition (9).

According to Gois et al. (31), depressive temperament may also be important to better understand the interplay between depression and diabetes.

Protective factors for suicidal risk were higher household income, higher education, social and emotional support (8, 12), and marriage or cohabitation. Being single was a risk factor for suicide (17); furthermore, suicide risk includes also gender, developmental, and substance abuse determinants (18, 21, 23). Recent studies have raised concern on the role of insulin and other antidiabetic medication use as a suicide method in patients with DM (21, 22). Having the means close at hand to commit suicide can represent a major risk for these patients.

Understanding the clinical features that can lead the patient with DM to have SI or suicidal behavior is an important objective to identify patients at higher risk and to promote well-being and adhering to antidiabetic medication. The present review of the relevant literature supports the need for further investigation into the severity and nature of disabilities associated with DM and other chronic diseases (32) and their relationships with SI and suicidal behaviors. From the clinical point of view, when treating patients with DM, it is important to emphasize that only a very small minority of patients eventually dies from suicide. However, given that DM *per se* is known to increase the risk of suicidal behavior and that depression is one of the most important single risk factors for suicide, it is important to be highly aware of co-occurring depressive symptoms in patients with DM.

These findings have implications for health-care professionals, pointing out the importance of adequate psychological screening and action plans for appropriate follow-up to reduce the suicide risk in diabetic patients.

## Author Contributions

All authors participated in the concept and writing of this manuscript; approved the final version of the manuscript.

## Conflict of Interest Statement

The authors declare that the research was conducted in the absence of any commercial or financial relationship that could be construed as a potential conflict of interest.

## References

[1] American Diabetes Association. Diabetes Care (2016) 39(Suppl 1):S1–2. 10.2337/dc16-S001 19118285 PMC2607066

[2] Ausili D Masotto M Dall’Ora C Salvini L Di Mauro S A literature review on self-care of chronic illness: definition, assessment and related outcomes (Una revisione della letteratura sul self-care nelle malattie croniche: definizione, valutazione e outcomes associati). Prof Inferm (2014) 67(3):180–9. 10.7429/pi.2014.673180 25392031

[3] Ali S Stone MA Peters JL Davies MJ Khunti K The prevalence of co-morbid depression in adults with type2 diabetes: a systematic review and meta-analysis. Diabet Med (2006) 23(11):1165–73. 10.1111/j.1464-5491.2006.01943.x 17054590

[4] Barnard KD Skinner TC Peveler R The prevalence of co-morbid depression in adults with type1 diabetes: systematic literature review. Diabet Med (2006) 23(4):445–8. 10.1111/j.1464-5491.2006.01814.x 16620276

[5] Pouwer F Nefs G Nouwen A. Adverse effects of depression on glycemic control and health outcomes in people with diabetes: a review. Endocrinol Metab Clin North Am (2013) 42(3):529–44. 10.1016/j.ecl.2013.05.002 24011885

[6] van Dooren FE Nefs G Schram MT Verhey FR Denollet J Pouwer F. Depression and risk of mortality in people with diabetes mellitus: a systematic review and meta-analysis. PLoS One (2013) 8(3):e57058. 10.1371/journal.pone.0057058 23472075 PMC3589463

[7] Goldston DB Kelley AE Reboussin DM Daniel SS Smith JA Schwartz RP Suicidal ideation and behavior and noncompliance with the medical regimen among diabetic adolescents. J Am Acad Child Adolesc Psychiatry (1997) 36(11):1528–36. 10.1016/S0890-8567(09)66561-8 9394937

[8] Amer NRY Hamdan-Mansour AM. Psychosocial predictors of suicidal ideation in patients diagnosed with chronic illnesses in Jordan. Issues Ment Health Nurs (2014) 35(11):864–71. 10.3109/01612840.2014.917752 25353299

[9] Pompili M Lester D Innamorati M De Pisa E Amore M Ferrara C Quality of life and suicide risk in patients with diabetes mellitus. Psychosomatics (2009) 50(1):16–23. 10.1176/appi.psy.50.1.16 19213968

[10] Fuller-Thomson E Sawyer JL. Lifetime prevalence of suicidal ideation in a representative sample of Canadians with type1 diabetes. Diabetes Res Clin Pract (2009) 83(1):9–11. 10.1016/j.diabres.2008.10.004 19070912

[11] Ceretta LB Réus GZ Abelaira HM Jornada LK Schwalm MT Hoepers NJ Increased prevalence of mood disorders and suicidal ideation in type2 diabetic patients. Acta Diabetol (2012) 49(1):227–34. 10.1007/s00592-012-0435-9 23064949

[12] Handley TE Ventura AD Browne JL Rich J Attia JR Reddy P Suicidal ideation reported by adults with type1 or type2 diabetes: results from diabetes MILES-Australia. Diabet Med (2015) 33(11):1582–9. 10.1111/dme.13022 26525943

[13] Chung JH Moon K Kim DH Min JW Kim TH Hwang HJ. Suicidal ideation and suicide attempts among diabetes mellitus: the Korea national health and nutrition examination survey (KNHANES IV, V) from 2007 to 2012. J Psychosom Res (2014) 77(6):457–61. 10.1016/j.jpsychores.2014.08.008 25258359

[14] Sarkar P Sattar FA Gode N Basannar DR. Failed suicide and deliberate self-harm: a need for specific nomenclature. Indian J Psychiatry (2006) 48(2):78. 10.4103/0019-5545.31594 20703390 PMC2913570

[15] World Health Organization. Public Health Action for the Prevention of Suicide: A Framework. Geneva: World Health Organization (2012).

[16] Liberati A Altman DG Tetzlaff J Mulrow C Gotzsche PC Ioannidis JP The PRISMA statement for reporting systematic reviews and meta-analyses of studies that evaluate health care interventions: explanation and elaboration. J Clin Epidemiol (2009) 62:e1–34. 10.1016/j.jclinepi.2009.06.006 19631507

[17] Lee HY Hahm MI Lee SG. Risk of suicidal ideation in diabetes varies by diabetes regimen, diabetes duration, and HbA1c level. J Psychosom Res (2014) 76(4):275–9. 10.1016/j.jpsychores.2014.02.003 24630176

[18] Roy A Roy M Janal M. Suicide attempts and ideation in African-American type1 diabetic patients. Psychiatry Res (2010) 179(1):53–6. 10.1016/j.psychres.2010.06.004 20630602

[19] Davis WA Starkstein SE Bruce DG Davis TME. Risk of suicide in Australian adults with diabetes: the Fremantle Diabetes Study. Intern Med J (2015) 45(9):976–80. 10.1111/imj.12860 26332624

[20] Westling S Ahrén B Träskman-Bendz L Westrin Å. High CSF-insulin in violent suicide attempters. Psychiatry Res (2004) 129(3):249–55. 10.1016/j.psychres.2004.09.004 15661318

[21] Löfman S Hakko H Mainio A Timonen M Räsänen P. Characteristics of suicide among diabetes patients: a population based study of suicide victims in Northern Finland. J Psychosom Res (2012) 73(4):268–71. 10.1016/j.jpsychores.2012.08.002 22980531

[22] Avci D Çetinkaya A Karahan S Oğuzhan N Karagöz H Başak M Suicide commitment with metformin: our experience with five cases. Ren Fail (2013) 35(6):863–5. 10.3109/0886022X.2013.801299 23742066

[23] Myers AK Grannemann BD Lingvay I Trivedi MH. Brief report: depression and history of suicide attempts in adults with new-onset type2 diabetes. Psychoneuroendocrinology (2013) 38(11):2810–4. 10.1016/j.psyneuen.2013.06.013 23978666

[24] Han SJ Kim HJ Choi YJ Lee KW Kim DJ. Increased risk of suicidal ideation in Korean adults with both diabetes and depression. Diabetes Res Clin Pract (2013) 101(3):14–7. 10.1016/j.diabres.2013.06.012 23871574

[25] Radobuljac MD Bratina NU Battelino T Tomori M Lifetime prevalence of suicidal and self-injurious behaviors in a representative cohort of Slovenian adolescents with type1 diabetes. Pediatr Diabetes (2009) 10(7):424–31. 10.1111/j.1399-5448.2009.00501.x 19490494

[26] Corathers SD Kichler J Jones NHY Houchen A Jolly M Morwessel N Improving depression screening for adolescents with type1 diabetes. Pediatrics (2013) 132(5):1395–402. 10.1542/peds.2013-0681 24127480

[27] Fornaro M Iovieno N Clementi N Boscaro M Paggi F Balercia G Diagnosis of co-morbid axis-I psychiatric disorders among women with newly diagnosed, untreated endocrine disorders. World J Biol Psychiatry (2010) 11(8):991–6. 10.3109/15622975.2010.491126 20569197

[28] Müller N Schwarz MJ. The immune-mediated alteration of serotonin and glutamate: towards an integrated view of depression. Mol Psychiatry (2007) 12(11):988–1000. 10.1038/sj.mp.4002006 17457312

[29] Martino M Rocchi G Escelsior A Fornaro M. Immunomodulation mechanism of antidepressants: interactions between serotonin/norepinephrine balance and Th1/Th2 balance. Curr Neuropharmacol (2012) 10(2):97–123. 10.2174/157015912800604542 23204981 PMC3386509

[30] Bai YM Su TP Chen MH Chen TJ Chang WH. Risk of developing diabetes mellitus and hyperlipidemia among patients with bipolar disorder, major depressive disorder, and schizophrenia: a 10-year nationwide population-based prospective cohort study. J Affect Disord (2013) 150(1):57–62. 10.1016/j.jad.2013.02.019 23510547

[31] Gois C Akiskal H Akiskal H Figueira ML. The relationship between temperament, diabetes and depression. J Affect Disord (2012) 142(Suppl):S67–71. 10.1016/S0165-0327(12)70010-1 23062859

[32] Conti C Carrozzino D Patierno C Vitacolonna E Fulcheri M. The clinical link between type D personality and diabetes. Front Psychiatry (2016) 7:113. 10.3389/fpsyt.2016.00113 27445869 PMC4914509

